# Skeletal muscle mass and adipose tissue alteration in critically ill patients

**DOI:** 10.1371/journal.pone.0216991

**Published:** 2019-06-13

**Authors:** Marie Mélody Dusseaux, Sami Antoun, Sébastien Grigioni, Gaétan Béduneau, Dorothée Carpentier, Christophe Girault, Steven Grange, Fabienne Tamion

**Affiliations:** 1 Department of Intensive Care, Rouen University Hospital, Rouen, France; 2 Gustave Roussy Institute, Emergency Unit, Villejuif, France; 3 Normandie University, UNIROUEN, Inserm U 1073, Rouen University Hospital, Rouen, France; 4 Normandie University, UNIROUEN, Inserm U 1096, Rouen University Hospital, Rouen, France; Universidade de Mogi das Cruzes, BRAZIL

## Abstract

**Background:**

Increasing numbers of studies in chronic diseases have been published showing the relationship between body composition (BC) parameters (i.e. skeletal muscle mass (SMM) and adipose tissue (AT)) and outcomes. For patients admitted to intensive care unit (ICU), BC parameters have rarely been described as a prognostic marker of outcome. The primary objective was to evaluate the relationship between body composition at ICU admission and major clinical outcomes. Secondary objectives were to assess the relationship between BC parameters and other parameters (systemic inflammatory markers, Sequential Organ Failure Assessment (SOFA) score, albumin level) at ICU admission, and between BC alterations during ICU stay and outcomes.

**Patients and methods:**

This retrospective study enrolled 25 adult patients who had two abdominal CT scans for clinical indication: first, within 48 hours of ICU admission (initial assessment), and second, 7 to 14 days later (late assessment). Skeletal Muscle radiodensity (SMD), cross-sectional area of SMM, Visceral Adipose Tissue (VAT) and Subcutaneous Adipose Tissue (SAT) were measured at the third lumbar vertebra. Cox regression analysis was used to determine the association between these parameters and mortality.

**Results:**

Patients’ mean age was 64.6 years. Their mean BMI was 27.7 kg/m^2^ (SD = 6.0). ICU mortality was 36%. There was no correlation between BC parameters at initial assessment and ICU outcomes. We observed a negative correlation between SMM index and SOFA score at initial assessment (r = -0.458, p = 0.037). There was a significant loss of VAT between two CT assessments which was associated with mortality (-22.34cm^2^ / m^2^ in non-survivors versus -6.22 cm^2^ / m^2^ in survivors, p = 0.039). Loss of SMD was greater with the occurrence of an infection than without (Delta SMD = -5.642 vs +1.957, p = 0.04).

**Conclusions:**

Our results show alterations in body composition during ICU stay with a loss of muscle quality (decreased SMD) and adipose tissue. These findings require confirmation in future studies but already show that BC assessments at ICU admission and BC alterations during ICU stay are important factors for outcome in critically ill patients.

## Introduction

Patients admitted to intensive care unit (ICU) are characterized by a systemic inflammatory response syndrome (SIRS), which triggers metabolic disorders. In critically ill patients, bed rest, systemic inflammation, and ICU-based insulin resistance contribute to loss of muscle. Skeletal muscle is a compartment of lean body mass and loss of muscle has been associated with longer duration of mechanical ventilation (MV) and higher ICU and hospital mortality [1–2]

Computed tomography (CT) analysis provides a unique approach for acquiring body composition measures and allows specific and precise, compartmentalized results for diverse lean and fat tissue [3–4]. CT analysis has been used to predict whole body muscle mass and to identify patients with low skeletal muscle mass (SMM) (i.e. sarcopenia) in cancer populations. In addition, loss in a patient’s physiological reserves and muscle depletion could be masked in overweight or obese patient. In a cohort of patients with non-small cell lung cancer, Baracos *et al*. showed a prevalence of sarcopenia of 47% whereas only 7.5% overall were underweight according to body mass index (BMI < 18.5) [5]. In another study, authors reported an association between body composition, especially sarcopenic obesity, assessed by CT in patients with solid tumors of the respiratory or gastrointestinal tract, and clinical implications as functional status and survival [6]. Since this leading article, many other studies in cancer patients have linked SMM depletion, independently of weight loss or BMI, to outcome in cancer patients [7]. In retrospective studies as many as 60–70% of patients had low muscle mass as assessed on CT scan at ICU admission, which was associated with higher mortality [8–9]

Not only the quantity, but also the quality of muscle as evaluated by skeletal muscle radiodensity (SMD), seems important [10–11]. Low skeletal muscle quality at ICU admission, as assessed by CT-derived SMD, is independently associated with higher 6-month mortality in mechanically ventilated patients [12]. Thus, muscle quality as well as muscle quantity are prognostic factors of outcome in ICU. This loss of SMM is linked to poor outcome not only for intra-hospital follow-up but also for long-term outcome. Survivors of acute respiratory distress syndrome (ARDS) have persistent functional disability one year after discharge from ICU with muscle wasting and weakness being most prominent [13]. Fatty infiltration of muscle has been identified as a possible cause of loss of muscle quality and CT scan can be used to measure SMD.

Changes in body composition parameters during hospitalization have been described as associated with outcomes in patients with cancer and other diseases. In patients with metastatic colorectal cancer, loss of muscle mass during chemotherapy was significantly associated with poor survival [14]. Puthucheary *et al*. reported a steady loss of SMM of almost 20% during the first 10 days of ICU [15].

The aim of the present study was to investigate the relationship between body composition as muscle quantity and quality, as assessed by CT, and major clinical outcomes as length of stay (LOS), duration of mechanical ventilation, nosocomial infection and mortality in critically ill patients. Second objectives were to evaluate the relationship between body composition changes during ICU stay and clinical outcomes.

## Patients and methods

This retrospective study enrolled critically ill patients admitted to the medical ICU of a university hospital from September 2011 to September 2013. CT-derived muscle analysis was performed at admission to ICU and 7 to 14 days after. The study has been approved by the institutional committee of Rouen University Hospital, Rouen, France (identification number E2014-29, 2014/12/11).

Patients were included if they were aged 18 years or older, in ICU for at least 7 days, required mechanical ventilation during their ICU stay, and had abdominal CT scans within the first 48 hours of admission to ICU (CT1: initial assessment) and 7 to 14 days after (CT2: late assessment CT2). These two CT scans were performed during ICU stay for clinical indication.

Patients were excluded if CT scans were not done within 48 hours of admission to ICU and 7 to 14 days after, or if data on body weight or height or Sequential Organ Failure Assessment (SOFA) scores were missing.

Demographic data parameters included: age, sex, weight, height, BMI, admission diagnosis, SOFA scores at ICU admission, serum albumin and serum procalcitonin (PCT) levels at initial and late assessment, the occurrence of a nosocomial infection, ICU-LOS, duration of MV, and mortality in ICU.

### CT scan analysis

Using standard operating procedures [3–4] data from lumbar cross-sectional areas (cm^2^) of SMM, visceral adipose tissue (VAT) and subcutaneous adipose tissue (SAT) were obtained. The third lumbar vertebra is commonly used as a bony landmark in most studies as it relates well to whole body SMM in healthy populations. Images were analyzed using Slice-O-Matic software V4_3 (Tomovision, Canada). Tissue was demarcated and quantified using Hounsfield unit (HU) thresholds of—29 to + 150 for skeletal muscles including the psoas, erector spinae, quadratus lumborum, transversus abdominus, external and internal obliques, and rectus abdominus, -150 to -50 for VAT, -190 to -30 for SAT.

These values were then normalized for height in square metres, expressed in units of cm^2^/m^2^ and reported respectively as the indices of SMM, VAT and SAT. Low SMM (i.e. sarcopenia) was defined according to SMM index thresholds (SMM index <55.4 cm2/m2 for men, < 38.9 cm^2^/m^2^ for women).

To evaluate the density of the skeletal muscle, we measured the mean radiation attenuation of skeletal muscle, which describes the input images read by the Slice-O-Matic software. The pixel values of these images displayed in shades of gray represent the physical properties of the scanned tissue expressed in numerical form. The mean attenuation (expressed as the mean HU) has been extensively studied as a correlate of muscle density. Muscle density assessed by this method reflects fatty muscle infiltration, with a lower mean HU indicating lower density and more fatty infiltration. This highly reproducible method correlates with muscle triglyceride contents on muscle biopsy [16].

All body composition (BC) measurements were performed by the same technician who was blinded to patient characteristics, clinical treatment and outcomes. Results are presented as index (units of cm^2^/m^2^), as expressed in oncologic studies. Other studies conducted in ICU report significant results on areas (units in cm^2^) [17].

## Statistical analysis

All statistical analysis was performed using SPSS 20.0 software (SPSS, Inc., Chicago, Illinois, USA). All measurement data are presented as mean +/- standard deviation (SD).

Descriptive statistics were computed separately for men and women for age, weight, height, BMI and SOFA score. The t-test was used because means were presented in normal distribution. The correlation between body composition at CT1 and clinical-biological parameters and then between body composition alterations and different outcomes (LOS, MV duration, nosocomial infection, death) was measured by Spearman correlation test. P< 0.05 was considered statistically significant. All p values given are two-tailed.

Logistic regression was performed to evaluate the independent determinants of mortality and acquired infection. Crude Odds ratios (OR) and adjusted Odds ratios (aOR) and their 95% confidence intervals (CI) were calculated.

## Results

### Patient characteristics

Twenty-five patients fulfilled inclusion criteria; their two CT scans were imported from the radiology system for analysis. Patient characteristics are presented in Table 1. Most patients were men (15/25). Mean age was 64.6 (± SD) years. Mean BMI was 27.7 kg/m^2^ (SD = 6.0). All patients had mechanical ventilation, with a mean time of 23.4 days (SD = 31.5). ICU mortality was 36% (9/25).

**Table 1:** Patient characteristics at admission (n = 25).

	Men (n = 15)	Women (n = 10)	p
Age, mean (SD), years	63.8 (10.99)	65.9 (12.95)	0.67
Weight, mean (SD), Kg	80.92 (15.5)	75.36 (20.5)	0.45
Height, mean (SD), meters	1.73 (0.06)	1.61 (0.039)	< 0.001
BMI, mean (SD), Kg/m2	26.89 (4.74)	28.94 (7.62)	0.41
SOFA score, mean (SD)	8.6 (4.53)	11.8 (4.63)	0.10
Serum albumin, mean (SD), g/L	25.06 (6.67)	21.12 (9.41)	0.27
PCT, mean (SD), ng/ml	52.48 (67.88)	28.40 (42.85)	0.35

SD = standard deviation, BMI = Body mass index, SOFA = Sequential Organ Failure Assessment, PCT = Procalcitonin

The causes of admission were: septic shock (n = 11), sepsis (n = 14) (acute severe pancreatitis (n = 6), cardiac arrest with inhalation (n = 2), pneumonia (n = 5), endocarditis (n = 1)).

Mean serum albumin level at admission was 23.37 g/L (SD = 7.99). Mean procalcitonin (PCT) levels at admission were 58.4 ng/ml in patients with septic shock and 21.56 ng/ml in other patients.

The mean number of days between CT1 and CT2 was 10.9 (SD = 4.7).

### Relationship between body composition at ICU admission and clinical-biological data

No significant correlation was observed between body composition at CT1 and ICU-LOS or mechanical ventilation duration [Table 2].

**Table 2:** Correlation between body composition at admission and clinical-biological parameters.

	Serum Albumin	p	SOFA Score	P	PCT CT1	p	LOS	p	MV duration	p
SMM index CT1	r = 0.720	0.001	r = -0.458	0.037	r = 0.235	0.305	r = 0.359	0.110	r = 0.208	0.366
VAT index CT1	r = 0.718	0.001	r = -0.055	0.813	r = 0.157	0.498	r = 0.251	0.272	r = 0.160	0.488
SAT index CT1	r = 0.345	0.207	r = 0.223	0.358	r = 0.026	0.917	r = -0.030	0.902	r = 0.042	0.864

PCT = Procalcitonin, CT1 = initial assessment, LOS = Length of stay in ICU, MV = Mechanical ventilation

SMM = Skeletal Muscle Mass, VAT = Visceral Adipose Tissue, SAT = Subcutaneous adipose tissue

r = Spearman correlation coefficient

There was a positive correlation (r = 0.720, p = 0.001) between serum albumin level at CT1 and SMM index, and a negative correlation (r = -0.458, p = 0.037) between SOFA score at CT1 and SMM index. If SOFA score at admission was high, muscle mass index was low.

### Body composition analysis at initial assessment and at late assessment and body composition alterations

Among the 25 patients included, alterations in body composition between CT1 and CT2 were evaluated in 21 patients for SMM, VAT, and SMD indices and in 16 patients for SAT index. CT analysis could not be performed for 4 patients due to image quality and difficulties to measure SMM and AT areas. There was no significant variation in the indices of SMM (-2.09 cm^2^/m^2^, p = 0.183) and SMD (-1.3 HU, p = 0.493) between CT1 and CT2. There was a significant decrease in VAT index between CT1 and CT2 (-12.36 cm^2^/m^2^, p = 0.005) [Table 3]

**Table 3:** Body composition alterations between initial assessment and late assessment (n = 21).

	CT1	CT2	Delta (CT2-CT1)	p
SMM index, mean (cm2/m2) (SD)	48.73 (12.57)	46.64 (10.85)	-2.09 (6.96)	0.183
SMD, mean (HU) (SD)	34.86 (10.46)	33.56 (7.67)	-1.30 (8.53)	0.493
VAT index, mean (cm2/m2) (SD)	57.33 (36.52)	44.97 (26.88)	-12.36 (17.72)	0.005
SAT index, mean (cm2/m2) (SD)	52.48 (37.72)	57.80 (40.27)	+5.30 (13.60)	0.140

SMM = Skeletal Muscle Mass; SMD = Skeletal Muscle Density, HU = Hounsfield Unit, VAT = Visceral Adipose Tissue, SAT = Subcutaneous Adipose Tissue, CT1 = initial assessment, CT2 = late assessment, SD = Standard deviation

#### Relationship between body composition alterations and clinical outcomes

No significant correlation was observed between body composition alterations (delta SMM, VAT or SAT) and ICU-LOS or mechanical ventilation duration.

Nine patients presented a nosocomial infection between CT1 and CT2. These patients had a loss of muscle density (-5.642 HU) compared to patients without nosocomial infection who had no loss of muscle density (+1.957 HU), (p = 0.04) [Table 4].

**Table 4:** Risk factors associated with nosocomial infection.

	Total	Nosocomial infection	No nosocomial infection	p	OR [95%CI]	ORa [95%CI]
Serum albumin	23.77 (7.16)	20.58 (4.43)	29.62 (7.84)	0.008	0.79 [0.62–0.98]	0.57[0.26–1.15]
SOFA score, mean (SD)	9.57 (4.90)	7.56 (4.19)	11.08 (4.99)	0.103	0.84 [0.68–1.04]	
PCT, mean (SD)	45.26 (65.47)	31.50 (76.41)	55.59 (57.24)	0.418	0.99 [0.98–1.01]	
Delta SMM index, mean (SD)	-2.09 (6.96)	-4.19 (6.77)	-0.52 (6.96)	0.241	0.92 [0.80–1.06]	
Delta VAT index, mean (SD))	-12.36(17.72)	-10.32 (18.49)	-13.88 (17.80)	0.660	1.01 [0.96–1.07]	
Delta SAT index, mean (SD)	+5.3 (13.6)	+5.2 (9.03)	+5.4 (16.98)	0.978	1.00 [0.93–1.08]	
Delta SMD, mean (SD)	-1.3 (8.53)	-5.642 (9,04)	1.957 (6.78)	0.046	0.88 [0.77–0.99]	0.71[0.45–1.10]

SD = standard deviation, SOFA = Sequential Organ Failure Assessment, PCT = Procalcitonin, SMM = Skeletal Muscle Mass; VAT = Visceral Adipose Tissue, SAT = Subcutaneous adipose Tissue, SMD = Skeletal Muscle Density

Delta VAT index was significantly higher in non-survivors than in survivors (- 22.34 cm^2^ / m^2^ versus—6.22 cm^2^ / m^2^, respectively, p = 0.039). Loss of VAT was greater in non-survivors than in survivors. Delta SAT index was positive in non-survivors and negative in survivors (+ 13.68 cm^2^ / m^2^ versus—1.21 cm^2^ / m^2^, respectively, p = 0.024). No significant correlation was observed between mortality outcome and SMM or SMD alteration [Table 5].

**Table 5:** Risk factors associated with mortality.

	Total	Non survivors	Survivors	p	OR [IC95%]	ORa [95%CI]
Serum albumin	23.77 (7.16)	25.01 (7.27)	22.67 (7.31)	0.518	1.05 [0.91–1.21]	
SOFAscore, mean(SD)	9.57 (4.90)	9.75 (5.78)	9.46 (4.52)	0.900	1.01 [0.84–1.22]	
PCT, mean (SD)	45.26 (65.47)	79.23 (85.89)	24.36 (40.00)	0.050	1.02 [1.00–1.03]	1.02 [0.99–1.05 ]
Delta SMM index, mean (SD)	-2.09 (6.96)	- 4.20 (5.87)	-0.80 (7.47)	0.289	0.93 [0.81–1.06]	
Delta VAT index, mean (SD))	-12.36 (17.72)	-22.34 (17.23)	-6.22 (15.6)	0.039	0.94 [0.87–0.99]	0.92 [0.85–1.02]
Delta SAT index, mean (SD)	5.31 (13.60)	+13.68 (16.95)	-1.21 (4.71)	0.024	1.21 [1.02–1.47]	1.28 [0.98–1.68]
Delta SMD, mean (SD)	-1.30 (8.53)	+2.72 (6,76)	-3.77 (8.78)	0.091	1.12 [0.98–1.28]	

SOFA = Sequential Organ Failure Assessment, PCT = Procalcitonin, SMM = Skeletal Muscle Mass; VAT = Visceral Adipose Tissue; SAT = Subcutaneous adipose tissue

## Discussion

In this retrospective study in critically ill patients, we have shown a relationship between a loss of muscle mass at admission and SOFA score. During hospitalization, the loss of skeletal muscle quality, as assessed by SMD, was associated with nosocomial infection and the loss of VAT with higher ICU mortality.

### The impact of skeletal muscle mass

Low muscle quantity, as assessed by skeletal muscle area on CT scans at ICU admission, is a risk factor for hospital mortality, independent of sex and APACHE II score. In our study, there was a correlation between SOFA score and SMM loss considering that SOFA score analyses and SMM loss were the result of proteolysis that occurred during the previous days before admission. In parallel, we observed a correlation between SMM index and serum albumin level at ICU admission (r = 0.720, p = 0.001). Serum albumin concentration is a remarkably strong prognostic indicator of morbidity and mortality in critically ill patients. Albumin concentration is no longer considered as a good marker of malnutrition [18] and few studies have correlated albumin with protein mass. Thus, the study of Cano *et al*. did not find any relationship between lean mass measured by bioelectrical impedance analysis (BIA) and albumin among chronic obstructive pulmonary disease (COPD) patients [19]. Kyle *et al*. found a correlation between lean mass, evaluated by BIA and albumin concentration (r = 0.216, p = 0.001), in 995 adults hospitalized in emergency [20]. The relationship observed in our study between muscle mass index at admission and serum albumin concentration could be interesting since albumin synthesis depends primarily on SMM loss and the availability of amino acids in previous days.

Critically ill patients can lose 17–30% of their muscle mass within the first 10 days of ICU admission. The magnitude of muscle wasting may be amplified in patients with multiorgan failure (16% loss in the first 7 days of ICU) compared with single-organ failure (3% loss in the first 7 days of ICU) [15]. Previous studies have demonstrated a relationship between skeletal muscle loss and survival, ICU-LOS, duration of MV and other clinical outcomes [21]. Braunschweig *et al*. evaluated the change in SMM, VAT, and inter muscular adipose tissue (IMAT) in 33 adults with acute respiratory failure. The overall decline in SMM averaged 0.49%/day between the 2 time points with no change in VAT or IMAT [17].

In a population of mechanically ventilated critically ill patients, low skeletal muscle area was observed in 63% of patients and was associated with higher mortality compared with control area [22]. Weijs *et al*. developed ICU-specific cut-off points for low muscularity and related these cut-off points to mortality (OR = 4.3, CI 2.0 to 9.0, p < 0.001). We found no correlation with mortality in contrast with the previous data. Muscle wasting occurring during critical illness has an impact on survival, and long-term functioning. Herridge *et al*. found functional disability in survivors of ARDS up to 5 years after admission to ICU [23] and Iwashyna *et al*. found functional limitations up to 8 years after severe sepsis [24]. We did not evaluate this point in our study.

### The impact of skeletal muscle density

In our study, the loss of muscle density during ICU stay increased significantly in the presence of a nosocomial infection. Decreased muscle density is dependent on a change in composition within the muscle fiber, secondary to fluid surcharge and an increase in the amount of fat [25]. The association between infection and decreased SMD is interesting since we found a higher level of PCT (ie a higher level of inflammation) in patients with infection. The link between inflammation and SMD has been widely described in oncology. Recently, a retrospective study including 491 mechanically ventilated critically ill adult patients with a CT scan obtained after ICU admission showed that low skeletal muscle quality was independently associated with higher 6-month mortality in mechanically ventilated patients [12]. Muscle density appears as a prognostic factor of survival in different studies in oncology. In 101 melanoma patients assessed by CT scan, a correlation was observed between muscle density and long-term progression-free survival and metastasis-free survival [26]. Similarly, in a population with adrenocortical carcinoma, worsened overall survival was correlated with decreased lean psoas muscle area and increased intra-abdominal fat [27] In a cohort of patients with metastatic renal cell carcinoma, authors suggested that high muscle density appears to be independently associated with improved outcome and could be integrated into the prognostic scores enhancing the management of these patients [28]. The mechanism between this myosteatosis and morbid-mortality remains unclear [29]. Insulin resistance, inflammation, oxidative stress and mitochondrial damage are involved in the relationship between the alteration of density and prognosis [30–31]. These mechanisms may be partially involved in sepsis with insulin resistance, in which the presence of fluid impregnation and fat infiltration leading to decreased muscle density could play a crucial pathogenic role and lead to a negative outcome. Also, we observed a negative correlation between Delta VAT index and Delta SMN index. Decreased muscle density is dependent on a change in composition within the muscle fiber, secondary to fluid surcharge and an increase in the amount of fat. The altered metabolic mechanisms associated with inactivity decrease the ability of muscles to oxidise lipids and promote a shift in muscle fuel utilisation from lipids towards glucose, causing accumulation of lipids in the muscle. These observations support the idea that fat infiltration is related to loss of muscle quality with inflammatory process. Our data may support this hypothesis. Nevertheless, fluid surcharge is a major factor in the loss of muscle quality and our study may not differentiate these two points.

### Adipose tissue alteration and outcomes

In our study, SAT and VAT seem to have a different impact on prognosis. We found that a loss of VAT had a negative relationship with outcome. Indeed, non-survivors had a significantly greater loss of visceral body fat than survivors. A decrease in VAT could signal accelerated lipolysis related to the severity of the inflammatory syndrome. Using post-mortem adipose tissue biopsies from critically ill patients, it has been suggested that changes in adipose tissue may render adipose tissue biologically active as a functional storage depot for potentially toxic metabolites, such as excess circulating glucose and triglycerides, thereby contributing to survival [32]. On the contrary, we found that a gain of SAT had a negative relationship with outcome. Differential metabolic activity may be a mechanism underlying differences in the metabolic activity of visceral and subcutaneous AT. Our study did not allow analysis of this difference.

### Limitations

Our study has several limitations. First, it is a retrospective study on few patients with heterogeneous pathologies and the complexity of critical illnesses may have led to residual confounding.Second, measures were extrapolated from a single-slice cross-sectional CT scan image: this technique assumes a linear relation between the different body components which can be subjected to many variations in ICU. Third, fluid surcharge is not totally differentiated using CT scan measures limiting the conclusions.

## Conclusions

In summary, we have shown an alteration in the quantity and quality of skeletal muscle in critically ill patients. We observed a negative correlation between skeletal muscle mass and SOFA score at ICU admission and we confirmed the relationship between the loss of adipose tissue and outcome. Therefore, muscle quality appears to be an equally important factor for outcome as muscle quantity and the assessment of body composition appears as a new approach in the management of critically ill patients. Future intervention studies should focus on preventing the alteration of muscle quantity, but also of muscle quality.
